# Using dynamic time warping for quantifying effects of sinusoidal oscillation deviations during EEG time series prediction and for finding interesting EEG and fMRI changes

**DOI:** 10.1186/1471-2202-16-S1-P63

**Published:** 2015-12-18

**Authors:** Dinov Martin

**Affiliations:** 1 Computational, Cognitive and Clinical Neuroimaging Laboratory, Imperial College London, W2 0NN, UK

## 

Dynamic time warping (DTW) has successfully been used in feature extraction and analysis of brain data [1,2]. However, DTW and other dynamic programming (DP) methods are not nearly as widespread in neuroscientific contexts, as they are in bioinformatics, where they are central [3]. DP algorithms are extremely powerful and efficient [4], so we explored further uses of DTW in the analysis of brain data. The first part a) applies DTW to successfully find locally interesting signal changes in EEG and functional magnetic resonance imaging (fMRI) data. In the second part b) we focused on harmonic analysis. Harmonic analysis as conducted via Fourier Transform (FT) or Wavelet Transform (WT) is extremely widespread in analyzing brain data, especially electroencephalography (EEG) data. The fundamental assumption of these methods is that a complex function (the time series waveform) can be represented as a superposition of sinusoidal oscillations. While comparing spectra is useful, it is incorrect to assume that the entirety of the spectral density is due to oscillatory activity proper (especially sinusoidal) [5]. We wanted to get some idea of the amount and effects of non-sinusoidal oscillations in EEG data.

In a) we quantify the amount of local signal dissimilarity, which is often equivalent to where there is interesting sudden activity, and in b) we quantify how much non-sinusoidal activity there is in spectra computed in a standard way. For a) we apply the method on a data set from a simultaneous EEG/fMRI session with 18 subjects that has been used in other published works before [6] and show that we can easily detect movement, changes in brain state accompanying large reaction time changes during a choice reaction time task, and other interesting events in the EEG and fMRI data sets. The method is applicable to both modalities, as DTW is very general and can be used with any type of time series. The method we used works by either running DTW on a selected time interval (or volume) against sequential intervals (or volumes), or by running it as a sliding-window for detecting sudden local signal changes in the EEG or fMRI. For b) we show that the spectral power, as it's typically computed, includes non-sinusoidal activity, when compared to the signal compared against a series of equal-length (to the signal) sinusoidal waves of varying frequencies. We also found when the sinusoidal-oscillation deviations occurred during an EEG time series extrapolation which we performed using a deep learning Toolbox. The EEG time series prediction gave another point of view for the oscillation changes. We find that the prediction breakdown points are related to sudden oscillation changes, which are presumably time points when other neural oscillator generators become active and mixed in the signal. We interpret both prediction breakdowns and momentary oscillatory deviations as being possible reflections of sudden phase changes in the local or global brain state related, which may be related to EEG microstate changes. We hope to bring to attention the flexibility and power of DP to the neuroscientific community.
